# Size matters: three methods for estimating nuclear size in mycorrhizal roots of *Medicago truncatula* by image analysis

**DOI:** 10.1186/s12870-019-1791-1

**Published:** 2019-05-04

**Authors:** Gennaro Carotenuto, Ivan Sciascia, Ludovica Oddi, Veronica Volpe, Andrea Genre

**Affiliations:** Department of Life Sciences and Systems Biology, University of Turin, 10125 Torino, Italy

**Keywords:** Endoreduplication, Ploidy, Arbuscular mycorrhiza, Confocal imaging, Image analysis

## Abstract

**Background:**

The intracellular accommodation of arbuscular mycorrhizal (AM) fungi involves a profound molecular reprogramming of the host cell architecture and metabolism, based on the activation of a symbiotic signaling pathway. In analogy with other plant biotrophs, AM fungi are reported to trigger cell cycle reactivation in their host tissues, possibly in support of the enhanced metabolic demand required for the symbiosis.

**Results:**

We here compare the efficiency of three Fiji/ImageJ image analysis plugins in localizing and quantifying the increase in nuclear size - a hallmark of recursive events of endoreduplication - in *M. truncatula* roots colonized by the AM fungus *Gigaspora margarita*.

All three approaches proved to be versatile and upgradeable, allowing the investigation of nuclear changes in a complex tissue; 3D Object Counter provided more detailed information than both TrackMate and Round Surface Detector plugins.

On this base we challenged 3D Object Counter with two case studies: verifying the lack of endoreduplication-triggering responses in *Medicago truncatula* mutants with a known non-symbiotic phenotype; and analysing the correlation in space and time between the induction of cortical cell division and endoreduplication upon AM colonization.

Both case studies revealed important biological aspects. Mutant phenotype analyses have demonstrated that the knock-out mutation of different key genes in the symbiotic signaling pathway block AM-associated endoreduplication. Furthermore, our data show that cell divisions occur during initial stages of root colonization and are followed by recursive activation of the endocycle in preparation for arbuscule accommodation.

**Conclusions:**

In conclusion, our results indicate 3D Object Counter as the best performing Fiji/ImageJ image analysis script in plant root thick sections and its application highlighted endoreduplication as a major feature of the AM pre-penetration response in root cortical cells.

**Electronic supplementary material:**

The online version of this article (10.1186/s12870-019-1791-1) contains supplementary material, which is available to authorized users.

## Background

Arbuscular mycorrhizal (AM) fungi are the most widespread plant symbionts [1, 2], belonging to subphylum Glomeromycotina, and colonize more than 80% of all terrestrial plant species, from liverworts to angiosperms [3]. The symbiotic interaction brings benefits to both partners: mycorrhizal fungi enhance their host plant fitness by facilitating water and mineral absorption [4], while plants feed their fungal symbionts with simple sugars (exoses) and lipids, derived from photosynthesis [5]. In response to reciprocal chemical exchanges between symbionts, a fungal hypha adheres to the root epidermis forming an hyphopodium. The signal-transduction pathway that prepares the host plant root to the correct association with AM fungi is known as Common Symbiotic Signalling Pathway (CSSP). In response to CSSP activation, at least one epidermal cell develops an intracellular accommodation structure, called the prepenetration apparatus (PPA), which anticipates hyphal colonization of both epidermal and cortical cells [6], where highly branched fungal structures - called arbuscules - develop [7]. As hyphae penetrate each host cell, an extension of the plant cell membrane assembles inside the PPA, creating a new cell compartment, the symbiotic interface [8], where intracellular hyphae and arbuscules are hosted [9].

**Fig. 1:**
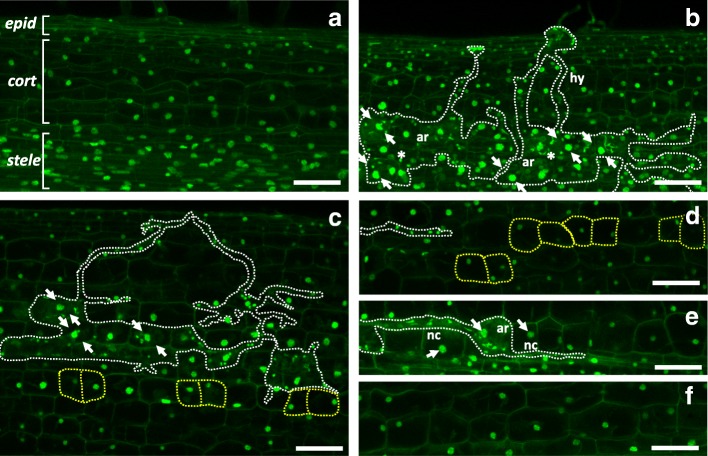
Increase in cell divisions and nuclear areas throughout AM colonization compared to uncolonized roots. The figure presents a collection of representative z-stack projections of uncolonized and mycorrhized wild-type *M. truncatula* ROCs. Panel (**a**) shows small sized nuclei in epidermal (epid), cortical (cort) and stele cells of an uncolonized root section. A colonized root section is shown in (**b**), where an Intraradical hypha (hy) of *G. margarita* (white dashed lines) penetrating from epidermal to cortical cells is visible, alongside several arbusculated cortical cells (ar). The latter display enlarged nuclei (white arrows), surrounded by small fungal nuclei (asterisks). Panels (**c**) and (**d**) show the appearance of ectopic cell divisions in the root cortex during progressive steps of AM colonization: couples of split cells (yellow dashed lines) are localized in proximity of arbuscules (**c**) and intraradical hyphae growing between cortical cells (**d**). The large nuclear size (arrows) in arbusculated and neighbouring cells (nc) is evident when compared to non-colonized cortical cells from the same root (panel e), as well as cortical cells from an uninoculated root (panel f). Scale bars = 50 μm

In this scenario, where fungal development must coordinate with host cell responses, PPA assembly is associated with complex activities of the plant cell nucleus, including its movement towards and away from the penetrating hyphal tip [6, 10]. In cortical cells, the eventual nuclear positioning at the cell center associates with a marked increase in size (Fig. 1) [11–16], representative of increased transcriptional, metabolic activities [10, 17] but also and above all endoreduplication onset, indicating host cell cycle machinery involvement. In fact, cell cycle reactivation in the root cortex in advance of fungal colonization is also demonstrated by the appearance of anticlinal cell divisions [18]. The resulting isolated couples of divided cells (here referred to as split cells) persist and are evident in longitudinal sections of the colonized area (Fig. 1 c, e) [19]. Indirect confirmation that ploidy increases are part of the AM accommodation program comes from gene expression increases in all major endoreduplication markers (such as *MtCCS52A* and *MtAPC subunit 2*) and from the analysis of mutant plant lines that cannot be colonized by AM fungi, where no change in nuclear size and ploidy are recorded [19].

In short, AM fungal accommodation associates with recursive activation of the cell cycle in the root cortex, leading to both cell division and multiple rounds of endoreduplication that eventually generate a mixed pattern of colonized and uncolonized cells displaying nuclei with diverse ploidy levels (ranging between 4 and 256C) and sizes [19].

The need to describe this cellular context prompted us to complement flow cytometry analyses of ploidy levels with different image analysis techniques, to get a more accurate picture of the relation between nuclear size and AM colonization in thick longitudinal sections of 4,6-diamidino-2-phenylindole (DAPI)-stained *Medicago truncatula* roots, imaged through confocal microscopy. To this aim, manual measurement of nuclear cross sections in confocal images has been used in the past [19], but the process is extremely time consuming. Scientific literature is rich in analogous cases, where the diameter, area or volume of discrete fluorescent objects has been estimated by automated or semi-automated image analysis methods [20–25]. Our images of plant root sections hosting fungal structures represented a challenging ground for image analysis software, mostly related to z-axis decrease in fluorescence intensity (due to sample thickness) and the additional staining of fungal nuclei and part of the plant cell walls. We therefore decided to test the effectiveness of three Fiji/ImageJ (http://imagej.nih.gov/;http://fiji.sc/Fiji) plugins in estimating nuclear cross section area and volume: TrackMate; 3D Object Counter; and a customized version of Fiji/ImageJ macros used for area estimation (here referred as Round Surface Detector). Our comparative analyses highlighted advantages and limitations of each approach, compared to manual measurement and indicated 3D Object counter as the most reliable automated method. We therefore decided to test it with two challenging case studies. Our results outline a remarkable correlation between calculated nuclear volumes and ploidy, and shed light on key aspects of fungal accommodation responses in AM interactions.

## Results

### Three-dimensional confocal images are suitable for the analysis of nuclear morphology

Our nuclear staining method was based on a protocol developed for analogous analyses of nuclear morphology in nematode-infected roots of *Arabidopsis thaliana* [22, 26] and modified according to Carotenuto et al. (2019) - to analyse *M. truncatula* root tissues engaged in AM. In particular, the use of DAPI staining on 100-μm thick Vibratome sections had previously proven successful in preserving root nuclei [22] and allowing detailed measurements of their areas, volumes and spatial distribution. In our samples (Fig. 1), plant nuclei were marked very brightly, with a high signal/noise ratio and weak non-specific labelling of cell walls. Importantly, DAPI also stained *Gigaspora margarita* nuclei (easy to discriminate from the plant nuclei based on their small size) in intraradical and extraradical hyphae.

**Fig. 2:**
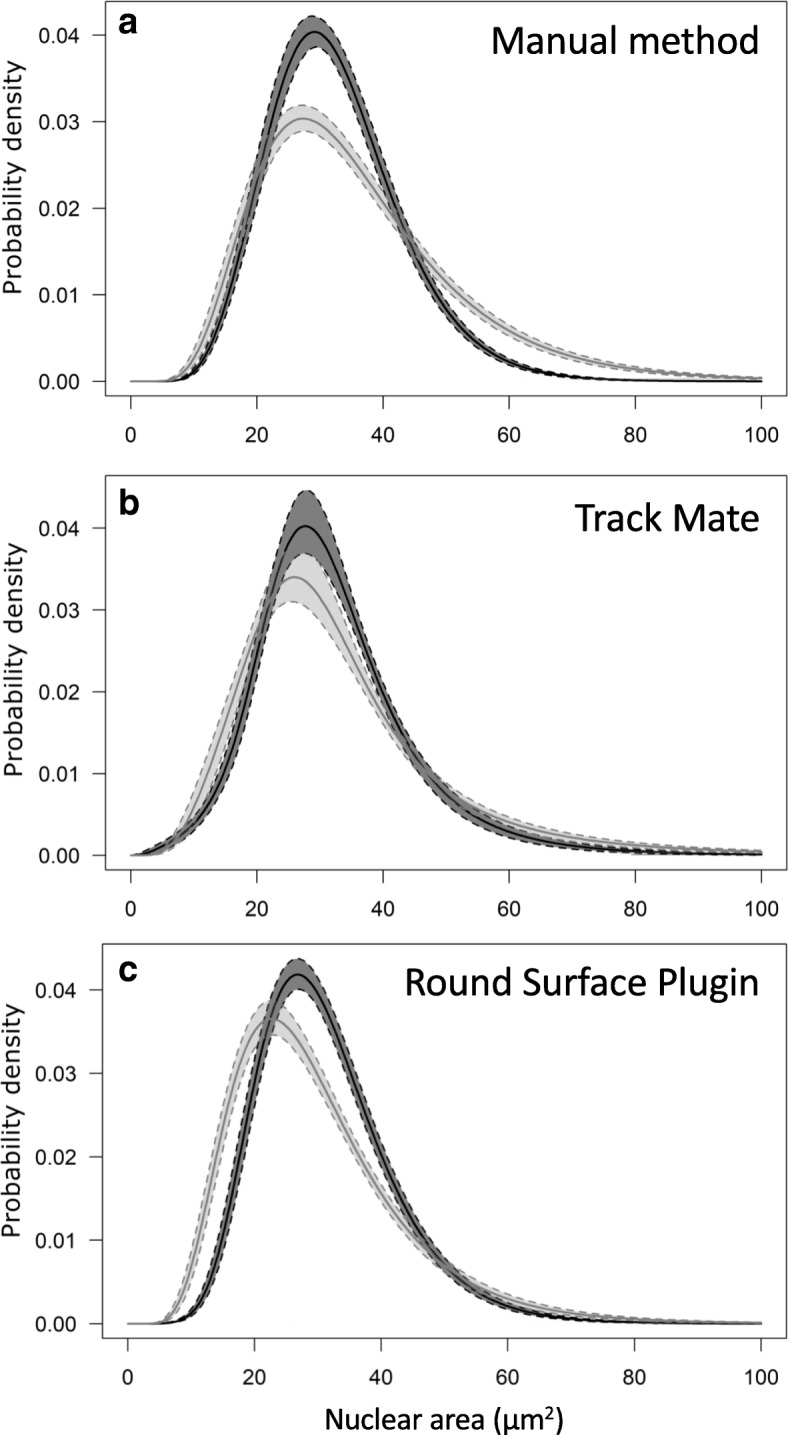
Lognormal distributions of nuclear areas in uninoculated and mycorrhizal roots of wild-type *M. truncatula.* The distribution curves of nuclear size, obtained through permutation analysis of areas detected by Manual method (**a**) showed significantly higher values of skewness (*P* < 0.001) and kurtosis (*P* < 0.01) in mycorrhizal (light grey) compared to uninoculated (dark grey) root segments. No statistically significant difference was observed between the distribution curves of nuclear areas estimated with TrackMate plugin (**b**). In analogy to the manual method, distribution curves of nuclear size detected by Round Surface plugin (**c**), showed significantly higher values of skewness (*P* < 0.001) and kurtosis (*P* < 0.01) in mycorrhizal compared to uninoculated root segments. Pairwise comparisons were performed with Tukey’s post-hoc test

In order to validate the results obtained through automated image analysis, the size of equatorial nuclear sections was first measured manually, as described in Carotenuto et al. (2019), by outlining each DAPI-stained nucleus in single optical sections from six z-stacks recorded in uninoculated and colonized roots. We measured an amount of 1020 and 1150 nuclei in uninoculated and colonized root sections respectively (Additional files 1, 9). Quantitative analysis showed that the average area of equatorial nuclear sections was significantly smaller (independent sample’s median test, *P* < 0.05) in uninoculated (32.7 ± 0.33 μm^2^) compared to mycorrhizal root segments (35.9 ± 0.48 μm^2^). In addition, the curve distribution of nuclear areas (Fig. 2a; Additional file 1) showed significantly higher values of skewness (*P* < 0.001) and kurtosis (*P* < 0.01) in mycorrhizal (ske = 1.14 ± 0.16; kur = 1.52 ± 0.49) compared to uninoculated root segments (ske = 0.74 ± 0.20; kur = 0.75 ± 0.56), due to the wider range of values observed in the former treatment (15–142 μm^2^) compared to the uninoculated one (15–62 μm^2^).

In conclusion, the manual measurement of nuclear cross sections in z-stacks from chemically fixed and DAPI-stained root segments highlighted a marked increase in nuclear size upon AM colonization, in line with our previous studies [19]. The image data set was therefore considered suitable for determining nuclear morphology by automated image analysis.

### TrackMate analysis of nuclear diameters proved limitations in estimation of cross section areas and volumes

TrackMate is a plugin originally designed to track cytoskeletal elements within time-lapse imaging of living cells [27]. In order to apply this software to our dataset, we chose to swap the time-axis with the z-axis, therefore forcing TrackMate to trace the selected objects (optical sections of DAPI-stained nuclei) across a confocal z-stack. The TrackMate detector automatically identified nuclear sections with coincident centroids, tagging them with a univocal ID and detecting their diameters, which were then used to calculate the cross-section area of individual nuclei. The resulting frequency distribution of nuclear cross-section areas showed an overall range increase between uninoculated (15–114 μm^2^) and mycorrhizal root sections (15–149 μm^2^), although no statistically significant difference between skewness and kurtosis values of the two curves was observed (Fig. 2c; Additional files 1, 9).

As a second step, assuming that nuclear sections could be approximated to circular objects, we used the TrackMate output data to estimate nuclear volumes based on confocal z-step and the diameters of all optical sections with the same ID tag (see Methods and Additional file 2).

**Fig. 3:**
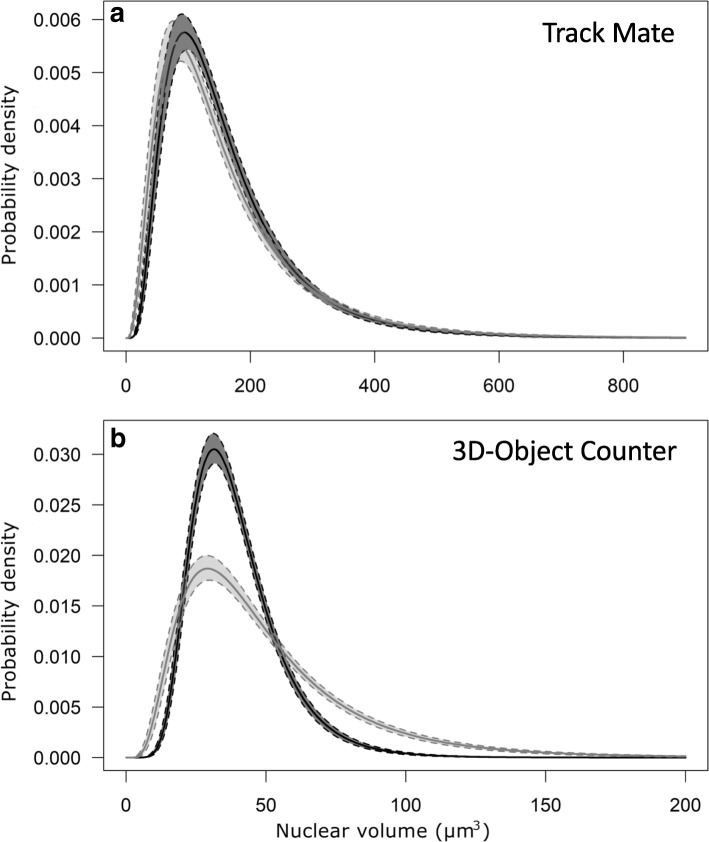
Lognormal distributions of nuclear volumes in uninoculated and mycorrhizal roots of wild-type *M. truncatula*. No statistically significant difference was observed between the distribution curves of nuclear volumes estimated with TrackMate plugin (**a**) in uninoculated (dark grey) and mycorrhizal (light grey) root segments. This result is indicative of the limited efficacy of this image analysis method. By contrast, the distribution curves, obtained through a bootstrap resampling method of volumes detected by 3D-Object Counter (**b**), revealed significantly higher values of skewness (*P* < 0.01), but not Kurtosis, in mycorrhizal compared to uninoculated roots. This indicates that 3D-Object Counter can be considered a more reliable method for this type of image analyses. Pairwise comparisons were performed with Tukey’s *post-hoc* test

Also in this case, the distribution curve of nuclear volumes (Fig. 3a; Additional file 1) showed no significant difference in skewness and kurtosis values between uninoculated (23–624 μm^3^) and mycorrhizal samples (23–891 μm^3^), although the occurring overall range increased.

Compared to manual measurements, our application of TrackMate to automatically detect and measure nuclear section diameters across z-stacks revealed critical limitations. In particular, our use of the TrackMate proved unreliable for nuclear size estimation from confocal z-stacks. Furthermore, the TrackMate analysis showed a low detection efficiency compared to the manual method: a marked number of cell nuclei remained undetected in both colonized (> 44%) and uninoculated (> 38%) roots.

### A customized plugin (round surface detector) efficiently identifies and measures nuclear cross-section

As an alternative to the previous approach, we wrote an image analysis algorithm using the Fiji embedded macro language (see Methods; Additional file 3; Additional file 4). The resulting plugin allowed automated nuclear tagging and identification based on roundness and the size range of the detected objects.

The detection efficiency of this Round Surface Detector plugin (Additional files 1, 9) produced results comparable to that achieved by manual measurement, identifying 1001 and 1147 nuclei in uninoculated and colonized root segments, respectively. In contrast to the manual method, no significant increase in average nuclear area between uninoculated and mycorrhizal root sections was detected by the Round Surface Detector, although the distribution range of nuclear cross section areas was considerably larger in mycorrhizal (15–142 μm^2^) than uninoculated roots (15–68 μm^2^). However, the curve distribution of nuclear areas (Fig. 2b) showed significantly higher values of skewness (*P* < 0.001) and kurtosis (*P* < 0.01) in mycorrhizal (ske = 1.37 ± 0.20; kur = 2.83 ± 0.91) compared to uninoculated root segments (ske = 1.04 ± 0.10; kur = 1.62 ± 0.52).

### Using 3D object counter for nuclear volume measurements

As the last method, we analysed our confocal datasets using 3D Object Counter, a Fiji plugin dedicated to 3D image analysis (see Methods; Additional file 5). This approach detected 594 and 893 nuclei in uninoculated and mycorrhizal roots, respectively, with a larger frequency distribution range (Additional files 1, 9) in mycorrhizal (20–220 μm^3^) than uninoculated roots (15–118 μm^3^). Compared to the manual approach, nuclear detection was less efficient (from 33% in colonized to 40% in uninoculated roots). This was probably caused by two problems within the algorithm. First, the selection of a single intensity threshold separating all nuclei from the background was difficult due to variations in staining intensity across the z-stack. Second, clustered nuclei - that were identified as a single lobed object - had to be manually deleted, thus reducing the total number of measurements.

Nevertheless, 3D Object Counter analysis highlighted a significant 1.25-fold increase (independent sample’s median test, *P* < 0.05) in average nuclear volume (Additional file 1) between uninoculated (40.3 ± 0.68 μm^3^) and mycorrhizal roots (50.7 ± 1.13 μm^3^), in line with manual measurements and previous studies [19]. The analysis of the distribution curve (Fig. 3b) confirmed this difference, showing significant higher values of skewness (*P* < 0.01) in mycorrhizal (1.47 ± 0.15) compared to uninoculated root segments (1.14 ± 0.22), whereas kurtosis value did not show any significant difference mainly because of the high variance.

In short 3D Object Counter resulted to be the most reliable method to automatically detect and measure nuclear size in mycorrhizal and uninoculated *M. truncatula* roots, representing a valid alternative to manual measurements. On this basis, we decided to apply this method to two specific and potentially challenging case studies.

### Case study 1: analysis of nuclear morphology in two mutant phenotypes

Based on our previous tests, we decided to use 3D Object Counter to compare nuclear volumes in the cortical tissue of uninoculated and inoculated roots from wild type and two mutants of *M. truncatula* (*dmi2–2* and *dmi3–1*). Such mutants are impaired for key CSSP genes: the receptor-like kinase DMI2 (for Does not Make Infection) and the nuclear calcium/calmodulin-dependent protein kinase (DMI3). Both strongly affect the development of functional AM symbiosis [28, 29], with an arrest of fungal colonization at the root epidermis, and a lack of cell cycle and endoreduplication reactivation in cortical cells [18, 19]. On this basis - and to prevent the abovementioned limitations of the 3D Object Counter algorithm - we restricted our measurements to the cortical tissue by cropping the original z-stacks (Additional files 6, 9).

**Fig. 4:**
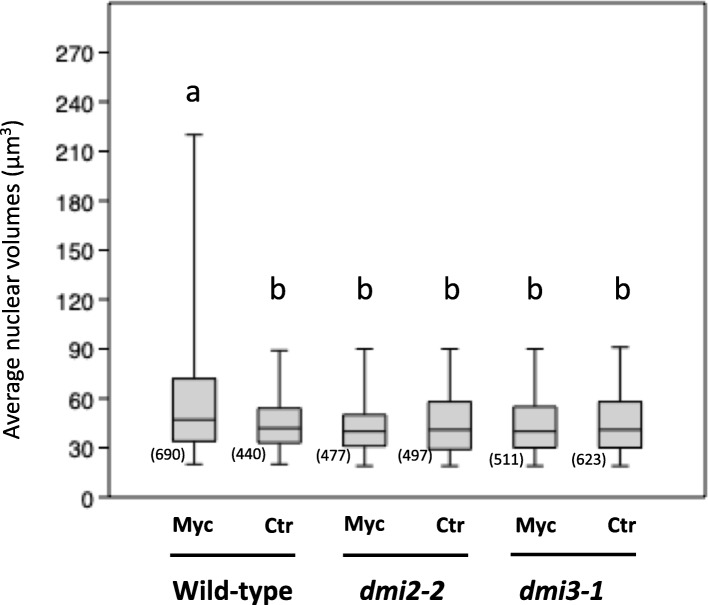
Average nuclear volumes of cortical cells in uninoculated and inoculated roots from wild-type, *dmi2–2* and *dmi3–1* ROCs. Measurements were performed using the Fiji plugin 3D Object Counter. Histograms report average volumes of DAPI-stained cortical nuclei in uninoculated and inoculated ROC segments from wild-type, *dmi2–2* and *dmi3–1 M. truncatula* ROC lines. Average nuclear volume is significantly increased in mycorrhizal compared to wild-type uninoculated cortex, as well as to uninoculated and inoculated cortical cells of *dmi2–2* and *dmi3–1* mutants. No statistically significant differences between samples was observed within the same lines. Bars represent standard deviations; letters indicate statstically significant differences based on Kruskal-Wallis non-parametric analysis of variance followed by Dunn’s post-hoc test (using Bonferroni-corrected *p*-values) for pairwise multiple comparisons (*P* < 0.05). Myc = inoculated; Ctr = uninoculated root cortex. The numbers in parentheses indicate the number of measurements for each experimental condition. The line inside each box represents their median, the top and bottom of each box represent upper and lower quartile respectively. Error bars represent standard deviation

A total of 440 and 690 nuclei were detected in uninoculated and mycorrhizal wild-type samples; 497 and 477 nuclei in uninoculated and inoculated *dmi2–2*; 623 and 511 nuclei, respectively, in *dmi3–1* (Fig. 4). The analysis indicated an average nuclear volume of 58.6 μm^3^ for cortical cells in colonized wild-type roots. By contrast, uninoculated wild-type and both uninoculated and inoculated *dmi2–2* and *dmi3–1* mutants displayed nuclei of approximatively 44 μm^3^ (Fig. 4).

While demonstrating the efficiency of the 3D Object Counter approach, this significant difference has relevant biological implications. The enlargement of cortical cell nuclei, recorded through manual measurement, had previously been related to AM colonization [19]. The present results - based on the use of an automated image analysis method and intrinsically excluding any possible bias related to manual measuring - confirm that this is a hallmark of AM fungal accommodation and is dependent on the functionality of the CSSP where DMI2 and DMI3 play key roles.

### Relationship between nuclear volume and endoreduplication

Increase in nuclear volume is the most obvious consequence of increments in ploidy [25, 30–32]. We therefore decided to check whether the population of nuclear volumes could be internally subdivided into a number of coherent classes, possibly reflecting progressive rounds of ploidy increase. To this aim, we applied the Sturges rule [33] to our dataset. The analysis, as reported in Additional file 7, indicated 25 μm^3^ as the optimal class width and identified four classes in the population of nuclear volumes from uninoculated wild-type roots, with average volumes of 30.3 μm^3^ for class I, 54.6 μm^3^ (class II), 77.6 μm^3^ (class III), 107 μm^3^ (class IV). By contrast, eight such classes were identified in mycorrhizal wild-type with average volumes of 29.6 μm^3^ (class I), 55.2 μm^3^ (class II), 81 μm^3^ (class III), 105.9 μm^3^ (class IV), 131.8 μm^3^ (class V), 155.3 μm^3^ (class VI), 181 μm^3^ (class VII) and 210.2 μm^3^ (class VIII).

Notably, the two populations of nuclear volumes – identified by applying descriptive statistics [33] to our dataset of measurements – matched with the corresponding ploidy levels and population of nuclear areas described by Carotenuto et al. (2019). On this basis, we used this clustering as an indication of putative ploidy levels, as shown in Additional file 8.

### Case study 2: cortical cell division vs ploidy increase in AM

In addition to the onset of diffuse and recursive endoreduplication events [19], the triggering of ectopic cell divisions, leading to the appearance of split cells (Fig. 1), has also been described in the AM colonized area of the root [18]. Such split cells are easily recognized for their roughly square shape [18], which is maintained over time due to the limited capability of wall extension of the fully differentiated surrounding tissue.

**Fig. 5:**
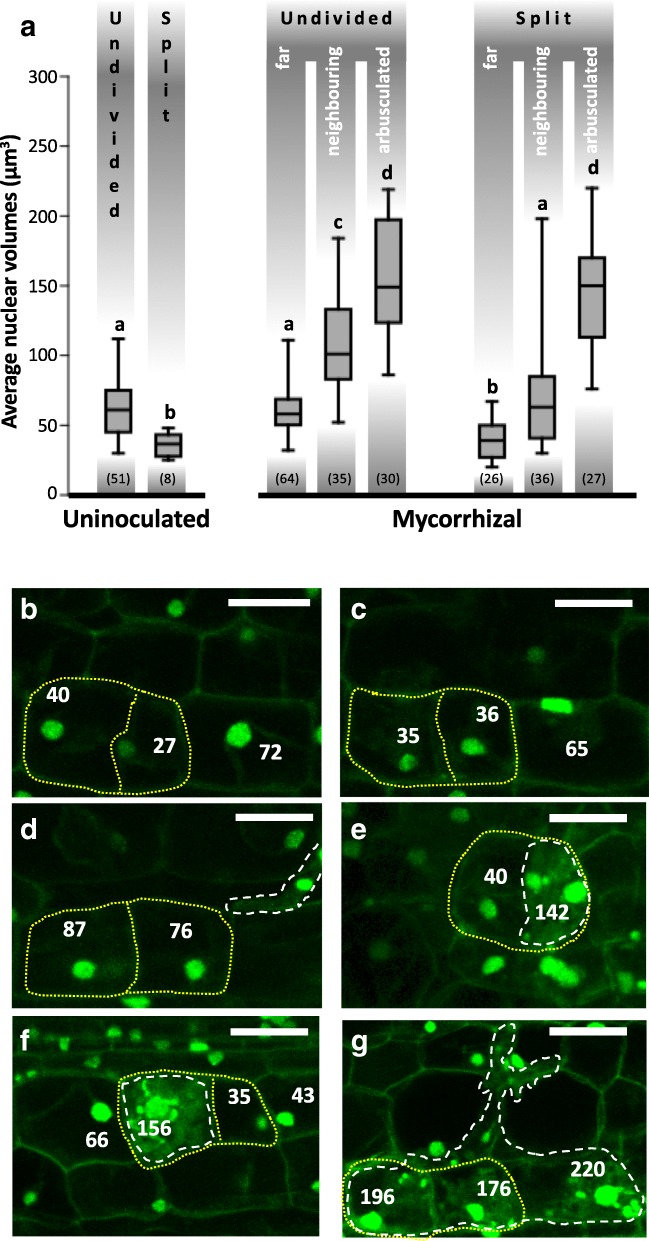
Increase in nuclear volumes follows cortical cell divisions throughout root colonization. **a** Average nuclear volume in non-divided and divided (here referred as split cells) cortical cells from uninoculated and mycorrhizal ROC segments of wild-type *M. truncatula*. For a more detailed representation of nuclear changes in wild-type mycorrhizal root cortex, we discriminated undivided and split cells in arbusculated from neighbouring and far uncolonized divided cells. Quantitative analysis was done on a dataset of 10 z-stacks from different uninoculated and mycorrhizal root segments. The graph shows a significant increase in average nuclear volume of arbusculated and uncolonized neighboring corticals in both undivided and split cells of mycorrhizal roots. The average nuclear volume of undivided corticals in uninoculated samples is comparable with that of far undivided cells of mycorrhizal roots. Also uncolonized split cells of uninoculated (split cells) and mycorrhizal (far split cells) sections show comparable average nuclear volumes. Bars represent standard deviations; letters indicate statstically significant differences based on Kruskal-Wallis non-parametric analysis of variance followed by Dunn’s post-hoc test (using Bonferroni-corrected *p*-values) for pairwise multiple comparisons (*P* < 0.05). The numbers in parentheses indicate the number of measurements for each experimental condition The figure presents a collection of representative z-stack projections of progressive steps in wild-type *M. truncatula* ROC colonization by *G. margarita* of cortical divided cells. Numbers indicate the volume of each nucleus in μm^3^. Panel (**b**) shows cortical divided cells (yellow dashed outline) in uninoculated root cortex with nuclei belonging to volume class I (20–45 μm^3^), as defined by Sturges’ rule. Panel (**c**) shows the same feature in split cells from inoculated roots far from the fungal penetration units. In (**d**), an Intraradical hypha (white dashed outline) colonizing the cortex is shown, with a couple of split cells containing class III nuclei (70–95 μm^3^), suggesting the occurrence of ectopic cell division (Russo et al., 2018) and endoreduplication (Carotenuto et al., 2019) before cell penetration. Panels (**e**) and (**f**) present early infection units, with couples of split cortical cells where only one cell is arbusculated. Such colonized cells display increasingly enlarged nuclei, with sizes reaching class V (120–145 μm^3^) in panel (e) and VI (145–170 μm^3^) in panel (f). More advanced infection units are shown in panel (**g**): large nuclei - class VII (170–195 μm^3^) and VIII (195–220 μm^3^) - are present in both split and undivided arbusculated cells. Bars = 25 μm.

Using the clustering of nuclear volumes into Sturges classes as an indication of putative ploidy levels, we focussed our analysis on all split cells found in our dataset of confocal z-stacks from uninoculated and mycorrhizal samples at progressive stages of fungal colonization (Fig. 5). Our aim here was to clarify how endoreduplication and cell division events combine during AM colonization. In fact, the progressive colonization of the root cortex from a single penetration point - characteristic of AM interactions [10] - allowed the observation of cell responses at different distances from intraradical hyphae, and provided a range of images depicting the whole progression of cell responses related to fungal accommodation.

Thus, nuclear volume measurements were extended to 10 uninoculated and 10 inoculated independent root sections of wild-type root organ culture (ROC) line (Fig. 5, Additional file 9). A total of 51 undivided and 8 split cortical cells were analyzed in uninoculated roots. The average nuclear volume in such undivided cells from uninoculated roots was 61 μm^3^ (Fig. 5a), corresponding to a putative 4C ploidy (Fig. 5b), as expected for this tissue, based on literature data [34, 35]. The few split cells observed in uninoculated samples displayed an average nuclear volume of about 36 μm^3^ (Fig. 5a, b), corresponding to putative 2C ploidy and suggesting that such cells originated from the division of a regular cortical cell.

In mycorrhizal *M. truncatula* roots we analyzed 129 undivided and 89 split cells (Fig. 5a). Among undivided cortical cells, 30 contained arbuscules (arbusculated), 35 were located next to an arbuscule (neighbouring) and 64 were at a distance of at least two cells from any arbuscule (far). Among split cells, we identified 27 arbusculated, 36 neighbouring and 26 far cells.

Far from the arbuscules (Fig. 5c), the situation was very similar to that of uninoculated roots: undivided far cells contained putative 4C nuclei (average nuclear volume of 61.1 μm^3^; Fig. 5a) while the nuclei of far split cells had an average volume of 38.9 μm^3^ (putative 2C ploidy).

By contrast, neighbouring cells displayed enlarged nuclei, with putative 16C nuclei (average of 110.1 μm^3^) in undivided cells and 8C nuclei (70.7 μm^3^ on average) in split cells (Fig. 5d). This suggested the occurrence of two endoreduplication rounds in each daughter cell.

Lastly, both undivided and split arbusculated cells (Fig. 5e-g) had nuclear volumes ranging between 8C and 128C (with an average nuclear volume of 157 μm^3^ for undivided and 143.7 μm^3^ for split cells; Fig. 5a). Remarkably, 13 cases (in a total of 81 z-stacks) were observed where only one of the split cells was hosting an arbuscule. Such images (exemplified in Fig. 5e, f) clearly show that the colonized cell hosts the largest nucleus. This provides convincing support to our current model of arbuscule accommodation (Carotenuto et al., 2019), where root colonization by AM fungi reactivates the cell cycle in the root cortex, initially leading to ectopic cell divisions and later to the onset of repeated endoreduplication rounds as cortical cells prepare to host an arbuscule.

## Discussion

### Validation of a reliable method to estimate nuclear size variations

Several methods have been developed to measure nuclear size during plant development [20, 23, 25] and biotrophic interactions [22, 36, 37]. Our previous analyses [19] showed how manual measurement of nuclear size in AM-colonized roots is laborious and strongly influenced by the staining method, prompting us to search for a sensitive, reliable and time saving alternative. By comparing the results of three automated or semi-automated image analysis methods based on Fiji software plugins and macros, we were able to select the algorithm that best matched the results obtained by manual analysis.

The “TrackMate” plugin was chosen for the versatility it showed when applied to protein dynamics, cell and organelle tracking [38–41]. After rerouting the TrackMate algorithm from time to z-axis in our confocal z-stacks, we were not able to detect the expected increase in nuclear area and volume in AM-colonized compared to uninoculated roots. The lack of any significant change in curve skewness and kurtosis, nor sample median, suggested that the geometrical approximation used to calculate nuclear area and volume from tracked diameters in serial optical sections was not reliable. TrackMate’s poor performance was likely due to the reduced fluorescence intensity in deeper optical sections and the progressive introduction of errors at each step of the formula used to estimate nuclear areas and volumes.

We therefore revised the TrackMate macro routines, using them to design the Round Surface Detector plugin for performing direct estimation of nuclear cross-section areas through different steps of image processing. The key requirement of this approach was the sectioning of each z-stack into thinner series of optical sections, to prevent the overlap of multiple nuclei with the same x-y coordinates. The Round Surface Detector plugin also involved a pre-emptive 3D rendering to more precisely outline the fluorescence halo of each nucleus. This approach revealed less error-prone and more performing that TrackMate, achieving a sensitivity in both nuclear tracking and surface estimation that was comparable to the manual method. In conclusion, the Round Surface Detector method is a promising replacement of the manual approach, even if an improvement of its 3D detection algorithm is advisable [21].

The best performance was obtained with the 3D Object Counter plugin. Even if the overall number of detected nuclei was lower than with Round Surface Detector plugin, the combination of thresholding, morphological segmentation and validation of the resulting map versus the original image allowed a very precise estimation of nuclear sizes. In fact, this method confirmed nuclear volume increase in AM-colonized compared to uninoculated and mutant root segments (case study 1), providing convincing indications that AM-associated ploidy changes are restricted to the root cortex [10, 19]. Furthermore, 3D Object Counter-based analyses shed new light on the relationship between cell division, nuclear size/ploidy increase and the progress of AM colonization (case study 2). It will now be interesting to test if a combination of algorithms can be designed to implement RDS detection efficiency (comparable to the manual method) with 3D Object Counter precision in volume estimation.

### A novel insight on host nuclear responses to AM colonization

Cell cycle is a central process in growth and development of multicellular organisms, including plants [42–44]. Following an initial phase of coordinated mitotic cycles, endoreduplication events are common in plant tissue differentiation, and are often essential for cell expansion and development of seeds, roots, hypocotyls and leaves [32, 45–48]. Furthermore, endoreduplication is also involved in physiological responses [45, 48], and responses to abiotic [34, 49–51] and biotic stimuli, such as pathogenic or symbiotic interactions [52, 53]. For instance, the occurrence of DNA double-strand breaks [49, 50] or gibberellin and ethylene biosynthesis [54, 55] can trigger endocycle activation. An increased ploidy level yields apparent advantages also under water [56] and light [57] deficient conditions. Lastly, endoreduplication can be triggered at pathogen infection sites, e.g. by nematodes [58], and upon the symbiotic interactions. Such manipulations of the host cell cycle have been thoroughly described in the mutualistic interaction between nitrogen-fixing rhizobia and legumes. Rhizobia have been shown to induce both mitosis and endoreduplication as a part of their infection process, enabling the development of new specialized organs (the root nodules) devoted to symbiotic nitrogen fixation [52, 53].

In AM, light microscopy, electron microscopy and morphometry have been employed to describe nuclear morphology in arbusculated cells [10, 11, 14] and our recent data indicate that fungal accommodation involves cell cycle reactivation in fully differentiated root cells, leading to localized cell divisions and endoreduplication [18, 19].

## Conclusions

Confocal microscopy and image analysis can be very helpful to interpret plant cell cycle reactivation during AM colonization. Nevertheless, such responses are particularly challenging to investigate, mostly due to the progressive and asynchronous process of root colonization by AM fungi, and none of the previous studies provided a detailed picture of the correlation between nuclear changes, cell divisions and progressive AM development.

Our application of an automated method to perform detailed image analysis of nuclear size, now extends our understanding of the relationship between cell division, nuclear ploidy and the progress of AM colonization in the cortical tissue. The necessity to clarify this elusive aspect had emerged in our previous studies, which documented how a combination of isolated cell divisions and ploidy increases in AM colonized areas results in a complex pattern of undivided and divided cells with different ploidy levels [18, 19]. Our novel results suggest that cell cycle reactivation during early root colonization by AM fungi triggers cell division in some cortical cells. Sustained cell cycle stimulation during later colonization of the inner root tissues leads to repeated rounds of endoreduplication, a cortical cell response that is now considered part of the arbuscule accommodation program and takes place in both undivided and split cortical cells (Carotenuto et al., 2019).

## Methods

### Plant and fungal materials

All experiments were done using explants of *Agrobacterium rhizogenes*-transformed root organ cultures (ROCs), derived from *M. truncatula* plants (genotype Jemalong A17). One ROC was derived from the wild-type cultivar and two from CSSP mutants *dmi2–2* and *dmi3–1*, [28, 29]. The ROC lines, available in the lab [59, 60], were prepared according to Russo et al. (2019) and Carotenuto et al. (2019).

The AM fungus *G. margarita* isolate BEG34 (International Bank for the Glomeromycota, University of Kent, Canterbury, UK) was used to colonize *M. truncatula* ROCs.

The targeted AM inoculation technique [61] was chosen to collect 1 cm-long root segments from uninoculated and early colonized roots (3 days after hyphopodium formation), as described in Carotenuto et al. (2019). Six independent samples were collected from independent uncolonized or colonized ROCs of wild-type and mutant lines, and prepared for confocal imaging.

### Confocal microscopy

Root segments were fixed in phosphate-buffered saline (PBS), pH 7.2, containing 1% formaldehyde and 10% dimethyl sulfoxide (DMSO) for 24 h at 4 °C, and treated as described in Carotenuto et al. (2019). In brief, after sectioning into 100 μm-thick sections using a Vibratome (Oxford Vibratome® sectioning system), nuclear staining was performed using the DNA-specific marker 4,6-diamidino-2-phenylindole (DAPI). Imaging was then performed with an upright Leica TCS SP2 confocal microscope fitted with a long distance 40X water-immersion objective (HCX Apo 0.80), using the 405 nm diode for DAPI excitation. All root sections were scanned at 400 Hz and 2X line averaged, generating 375 × 375x45μm z stacks (z step = 1.5 μm) to be used for image analyses. Nuclear coss-section areas and volumes were measured using each of the following methods in a set of confocal images recorded from six independent root samples harvested for each experimental condition. Maximum brightness projections were generated for illustrative figures.

### Nuclear cross-section area and volume measurements using TrackMate

Image z-stacks of DAPI-stained root sections of *M. truncatula* were analysed with TrackMate, an interactive Fiji plugin including a wizard-like graphical user interface, originally developed for detecting and tracking objects in time-lapse image stacks [62]. For our image dataset, tracking was carried out by swapping the detection from time-axis to z-axis. Firstly, a spatial calibration of the z-stack was made, based on pixel size (1024 × 1024 pixels = 375 × 375 μm) and z step (1.5 μm) between optical sections.

The TrackMate detector was then programmed to use the ‘Laplacian of Gaussian*’* filter with a ‘blob estimated diameter’ set to 10 μm, fitting with the maximum size of *M. truncatula* nuclei; the Trackmate ‘quality feature’ was set to select only the brightest nuclei among the detected blobs; lastly, the ‘simple lap tracker’ was configured to tag with univocal IDs all nuclear spots sharing the same x-y centroid position across the z-stack. Diameters of all selected blobs were then calculated in each optical section.

The resulting dataset of ID-tagged nuclear diameters was exported to SQL to identify the largest diameter (d_MAX_) for each ID and use it to estimate the equatorial plane area for each nucleus (cross-section area) as π•(d_MAX_/2)^2^. Secondly, the same approach was used to approximately estimate nuclear volumes: the areas of all nuclear sections with the same ID tag was calculated, multiplied by the z-step (1.5 μm) to obtain partial volumes per each optical section (Additional file 2), and summed as $\sum \limits_{i=1}^n\left[\left(\Pi \bullet {\left(\frac{\mathrm{di}}{2}\right)}^2\right)\bullet 1.5\right]$. Only nuclear IDs recorded in at least three optical sections were used to estimate nuclear volumes, in order to exclude incompletely-scanned nuclei at the top or bottom ends of the z-stack.

### Nuclear cross-section area measurements using round surface detector

Using the Fiji macro language, we designed a plugin to automatically measure nuclear cross-section areas (Additional file 4). This plugin operates on confocal z-stacks through a routine of image analysis steps (Additional file 3).

First, z-stacks are calibrated based on pixel dimensions and z-step, as described for the TrackMate method. Optical sections from each z-stack are then rendered into a single 3D image using the ‘3D project’ function with the ‘brightest point’ method. The resulting 3D image is corrected for brightness and contrast to achieve maximal dynamic range, cleaned by ‘subtraction of background’, thresholded and masked using the ‘convert to mask’ function. The mask is then adjusted with ‘smooth’, ‘fill holes’ and ‘watershed’ operations to precisely map the three-dimensional outline of each identified object (nuclei and cell wall clusters). The mapped 3D image is further elaborated using the ‘analyse particles’ function with a high circularity index (ranging from 0.7 to 1) to exclude wall clusters and tag each nucleus with a univocal ID. The size of detected objects is conditioned by a user-selected range (from 15 to 150 μm^2^), in order to exclude smaller fungal nuclei and non-specific staining of plant cell walls. The final output can be viewed on screen and manually inspected to check for and discard possible mismatches.

### Nuclear volume evaluation using 3D object counter

The same image dataset was also analysed using the Fiji 3D Object Counter plugin for volume estimation from confocal z-stacks [63]. In addition, 3D Object Counter analysis was extended to six uninoculated and six inoculated independent root sections from both *dmi2–2* and *dmi3–1* mutants. Following spatial calibration and brightness/contrast adjustment, a threshold was applied to discriminate between the brighter nuclear voxels and the darker background [21]. The 3D Object Counter plugin was then used to generate a 3D surface map containing only nuclear voxels and sum them to estimate the volume of each nucleus.

Lastly, we merged the original z-stacks with the 3D Object Counter-generated surface maps to identify and tag each nucleus (Additional file 5) for a final visual control.

### Statistics and data analysis

Since the data did not fit a normal distribution (Shapiro-Wilk test, *P* < 0.05), the differences in the median values and frequency distributions between uninoculated and colonized wild-type root segments were compared using the non-parametric independent sample median test. Moreover, data distribution across the dimensional range was investigated separately for nuclear area and volume, prior to test the differences among treatments. Gamma and lognormal distribution were selected as best candidates to describe nuclear dimension distribution, and thus tested for their goodness of fit. Lognormal distribution showed the highest fit for all the data-samples, according to both Anderson-Darling statistics and Akaike’s Information Criterion. In order to homogenise the sample size of our dataset and make the comparison among treatments and methods more robust, a bootstrap resampling method with 1000 iterations was performed for each sample. The sample size was set to 500, and a lognormal curve was fitted to every re-sampled dataset to obtain the density values. Finally 500 random points were generated for each starting sample according to the density values obtained in the previous step, and the first moment-statistics of the new data-sample were estimated (i.e. mean, standard deviation, CV, skewness, and kurtosis). Generalised Linear Models (GLMs) of skewness and kurtosis statistics were used to investigate the differences between controls and mycorrhized samples detected with the abovementioned methods (i.e. Manual, TrackMate, Round Surface Detector, 3D Object Counter). Indeed, these moment-statistics, rather than mean and standard deviation, allowed us to focus on the effects of different probability density occurring at the curve tails, by describing the curve symmetry (i.e. skewness) and ‘tailedness’ (i.e. kurtosis). Pairwise comparisons were performed with Tukey’s post-hoc test. Before fitting the GLMs, response variables were tested for normality with Shapiro-Wilk test [64], and transformed when necessary (square root transformation). All the statistical analyses and plots were performed using both Past multivariate statistics software package v3.0 [63] and the computing environment R 3.5.1 (R Development Core Team, 2005) with the following packages: ‘dplyr’, ‘fitdistrplus’, ‘ggplot2’, ‘logspline’, ‘multcomp’, ‘nsRFA’, and ‘Rmisc’.

Regarding the cases of study, the statistical differences in the average nuclear volumes between uninoculated and inoculated roots of different ROC lines (i.e. wild-type, *dmi2–2* and *dmi3–1*) were evaluated with Kruskal-Wallis non-parametric analysis of variance followed by Dunn’s post-hoc test (using Bonferroni-corrected *p-values*) for pairwise multiple comparisons, accepting significant differences at *P* < 0.05 (Figs. 4 and 5). The volume dataset was further analysed using the Sturges rule [19, 33], a statistical method that allocates data into an optimal number of classes calculated with the following formula based on population characteristics:

$$ \frac{R}{1+3.322\ \mathit{\log}\ N} $$

where *R* is the range of a statistical series and *N* the number of items in the dataset (Additional file 7). These analyses were performed using both Past software package v3.0 and Excel.

## Additional files

Additional file 1: Descriptive statistics of nuclear areas and volumes measured by manual, TrackMate, Round Surface Detector and 3D Object Counter methods in mycorrhizal (Myc) and uninoculated (Ctr) root segments of wild-type *M. truncatula*; skewness and kurtosis values of the distribution curves obtained by a bootstrap resampling method of the nuclear areas and volumes detected by the different methods (PDF 46 kb)

Additional file 2:Schematic illustration showing how nuclear area and volume are detected by TrackMate method. (a) The nuclear blobs are tracked across the z-stack and tagged with univocal IDs. (b) All IDs associated with their diameters (red dashed lines) - sharing the same x-y centroid coordinates - are selected using an SQL analysis. The correspondent tridimensional image is created with Fiji 3D surface plot plugin. (c) The largest diameter (d_MAX_) was identified for each ID-tagged nucleus and used to estimate the equatorial plane area as π•(d_MAX_/2)^2^. (d) Based on the z-stack slice interval (1.5 μm, violet double arrow), nuclear volumes were calculated as $\sum \limits_{i=1}^n\left[\left(\Pi \bullet {\left(\frac{\mathrm{di}}{2}\right)}^2\right)\bullet 1.5\right]$. Scale bars = 50 μm (a,b) 20 μm (c) (PDF 972 kb)

Additional file 3: Schematic illustration showing how nuclei are detected by the automated digital image analysis method (Round Surface Detector). (a) Original DAPI-stained image is spatially calibrated. (b) Result of 8-bit conversion followed by adjustement of brightness and contrast of each frame with final 3D rendering. (c) Result after the increase of image quality by subtracting background and calibrating the threshold in order to obtain a mask. (d) Mask based on threshold is before blurred with a smooth (e) then adjusted by filling holes and separating linked objects with a watershed in order to define the nuclear borders. (f) Selection from E loaded into the original image where the surfaces are measured using a high circularity index (0,7–1). Scale bars = 50 μm (PDF 1789 kb)

Additional file 4: Round Surface Detector plugin for Fiji (TXT 1 kb)

Additional file 5: Schematic illustration showing how nuclear volumes are detected using 3D Object Counter. (a) Original DAPI-stained image is spatially calibrated and adjusted in brightness and contrast. (b) A threshold was applied to define the limit intensity value separating the voxels between background voxels (intensities below the selected value) and objects voxels. (c) A 3D surface map containing only nuclear voxels is generated and the volume of each nucleus is calculated. (d) A merge between original z-stacks and 3D surface map is created. Scale bars = 50 μm (PDF 2518 kb)

Additional file 6: Nuclear volume analysis of *M. truncatula* root cortex. Schematic illustration showing how nuclear volume detection is restricted to the cortical tissue by cropping (red box and right panel) the original z-stacks (left panel). The image dataset was then analysed using Fiji plugin 3D Object Counter, discriminating between the brighter nuclear voxels and the darker background. Scale bars = 50 μm (PDF 954 kb)

Additional file 7: Putative ploidy classes based on Sturges analysis of nuclear volume measures. All nuclear volumes measured with 3D Object Counter in 375x375x45μm z-stack projections from *M. truncatula* uninoculated (6 root sections, 594 nuclei) and mycorrhizal (6 root sections, 893 nuclei) ROC segments were clustered according to Sturges’ rule into 25 μm^3^ wide classes. Four classes were identified in uninoculated and eight in mycorrhizal sections; size limits and average volume ± standard deviation are presented for each class (PDF 29 kb)

Additional file 8: Maps of putative ploidy in the cortex of uninoculated and mycorrhizal roots of *M. truncatula*. Based on the Sturges rule analysis of all nuclear volume measures derived by 3D Object Counter (Additional file 7), four and eight ploidy classes were respectively identified in uninoculated (a) and mycorrhizal (b) sections. Panels show representative images. Scale bars = 50 μm (PDF 671 kb)

Additional file 9: Dataset of nuclear size measurements obtained with the different methods and for each case study. Sheet 1 and 2 of the Excel file include all measurements of nuclear areas and volumes (respectively), obtained with manual and automated analyses of confocal images from uninoculated and mycorrhizal root segments. The dataset deriving from the two case studies are presented in sheets 3 and 4 (XLSX 71 kb)

## Abbreviations

AM: Arbuscular mycorrhiza
CSSP: Common symbiotic signalling pathway
DAPI: 4,6-diamidino-2-phenylindole
PPA: Prepenetration apparatus
ROC: Root organ culture
