# Supplementary material for: Size matters: three methods for estimating nuclear size in mycorrhizal roots of Medicago truncatula by image analysis
Source: BMC Plant Biol. 2019 May 4;19:180. doi: 10.1186/s12870-019-1791-1 (PMC6500585; doi:10.1186/s12870-019-1791-1)
Supplement: Supplementary file 1 — Descriptive statistics of nuclear areas and volumes measured by manual, TrackMate, Round Surface Detector and 3D Object Counter methods in mycorrhizal (Myc) and uninoculated (Ctr) root segments of wild-type M. truncatula; skewness and kurtosis values of the distribution curves obtained by a bootstrap resampling method of the nuclear areas and volumes detected by the different methods (PDF 46 kb) [file 12870_2019_1791_MOESM1_ESM.pdf]

	Manual measurements		TrackMate surface		RSD		TrackMate Volume		3D-OC	
	**Myc**	**Ctr**	**Myc**	**Ctr**	**Myc**	**Ctr**	**Myc**	**Ctr**	**Myc**	**Ctr**
**Sample size**	1150	1020	628	634	1147	1001	552	634	893	594
**Min. value**	15	15	15	15	15	15	23	23	20	20
**Max. value**	142	62	149	114	142	68	890	768	220	118
**Average**	35.9 μm^2	32.7 μm^2	34.4 μm^2	32.4 μm^2	29.7 μm^2	31.2 μm^2	171.3 μm^3	165.8 μm^3	50.7 μm^3	40.3 μm^3
**Median**	33 μm^2	32 μm^2	29 μm^2	30 μm^2	26 μm^2	30 μm^2	140.5 μm^3	141.5 μm^3	39 μm^3	36 μm^3
**Stand. Dev.**	16.5	10.7	19.3	12.7	14.2	10.8	132	111.8	33.8	16.8
**Stand. Er.**	0.48	0.33	0.76	0.5	0.42	0.34	5.6	4.4	1.13	0.68
**Skewness**	1.14 ± 0.16	0.74 ± 0.20	1.03 ± 0.20	0.98 ± 0.33	1.37 ± 0.20	1.04 ± 0.10	1.43 ± 0.31	1.36 ± 0.18	1.47 ± 0.15	1.14 ± 0.22
**Kurtosis**	1.52 ± 0.49	0.75 ± 0.56	1.26 ± 0.71	1.30 ± 1.14	2.83 ± 0.91	1.62 ± 0.52	2.93 ± 1.72	2.78 ± 1.05	2.62 ± 0.86	2.14 ± 0.96
