# Supplementary figures and images for: Size matters: three methods for estimating nuclear size in mycorrhizal roots of Medicago truncatula by image analysis
Source: BMC Plant Biol. 2019 May 4;19:180. doi: 10.1186/s12870-019-1791-1 (PMC6500585; doi:10.1186/s12870-019-1791-1)

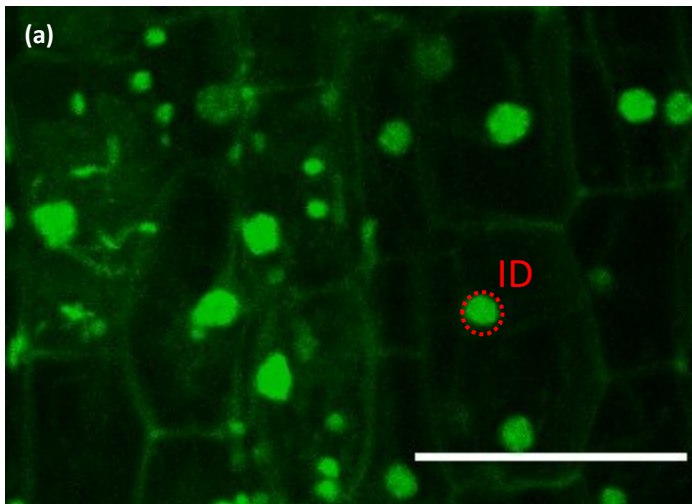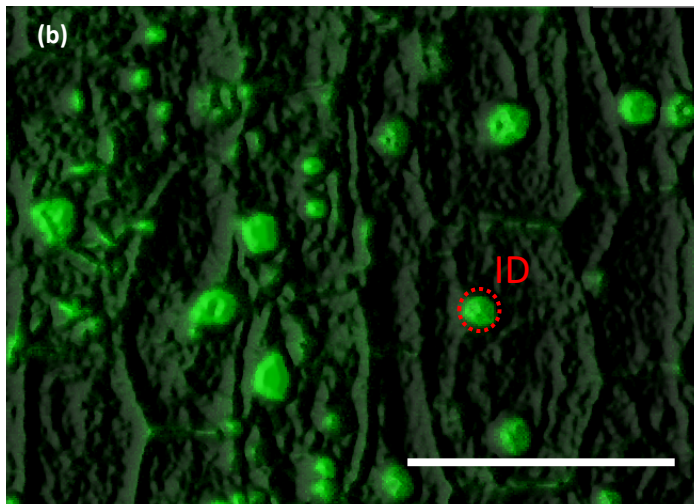

(c) (d)

$$\pi \cdot (d_{\text{MAX}}/2)^2$$

$$\sum_{i=1}^n \left[ \left( \pi \cdot \left( \frac{d_i}{2} \right)^2 \right) \cdot 1.5 \right]$$

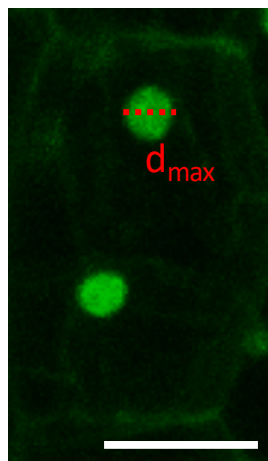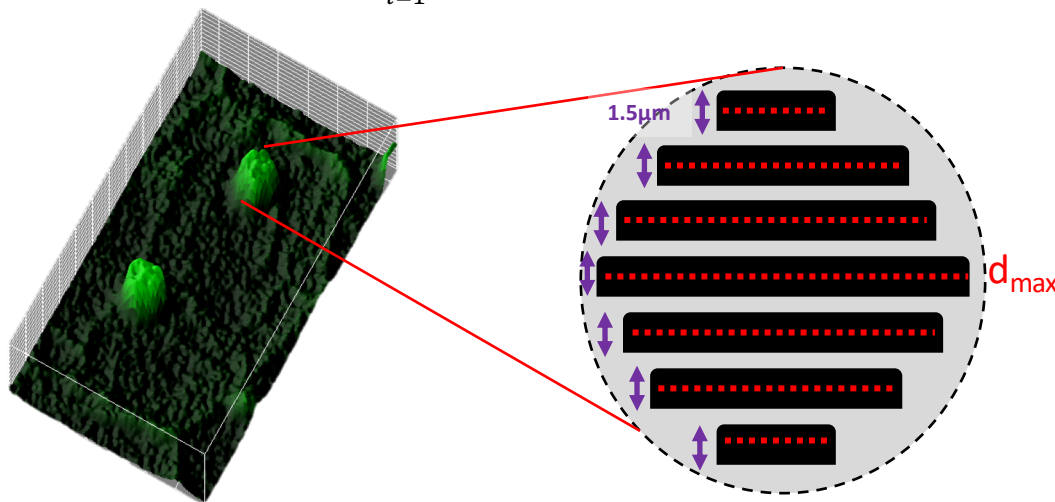

Supplement: Supplementary file 2 — Schematic illustration showing how nuclear area and volume are detected by TrackMate method. (a) The nuclear blobs are tracked across the z-stack and tagged with univocal IDs. (b) All IDs associated with their diameters (red dashed lines) - sharing the same x-y centroid coordinates - are selected using an SQL analysis. The correspondent tridimensional image is created with Fiji 3D surface plot plugin. (c) The largest diameter (dMAX) was identified for each ID-tagged nucleus and used to estimate the equatorial plane area as π•(dMAX/2)2. (d) Based on the z-stack slice interval (1.5 μm, violet double arrow), nuclear volumes were calculated as $\sum \limits_{i=1}^n\left[\left(\Pi \bullet {\left(\frac{\mathrm{di}}{2}\right)}^2\right)\bullet 1.5\right]$∑i=1nΠ•di22•1.5. Scale bars = 50 μm (a,b) 20 μm (c) (PDF 972 kb) [file 12870_2019_1791_MOESM2_ESM.pdf]

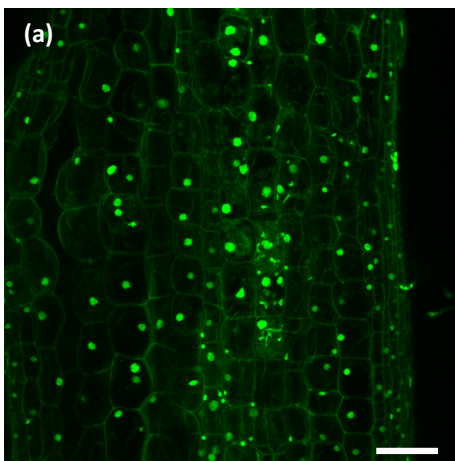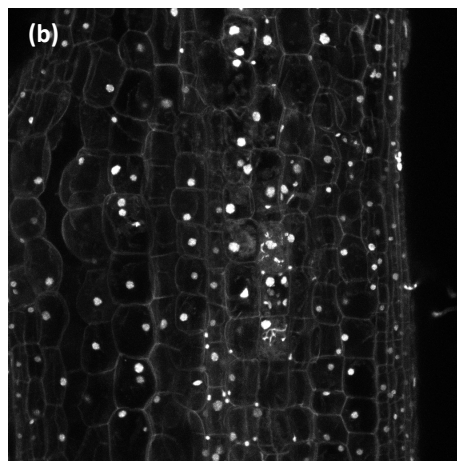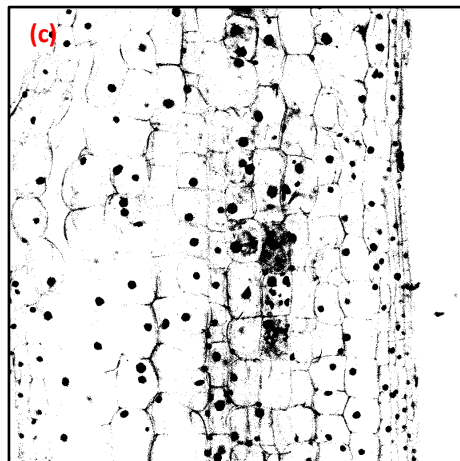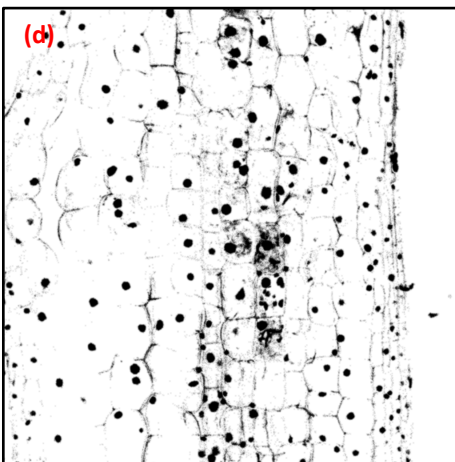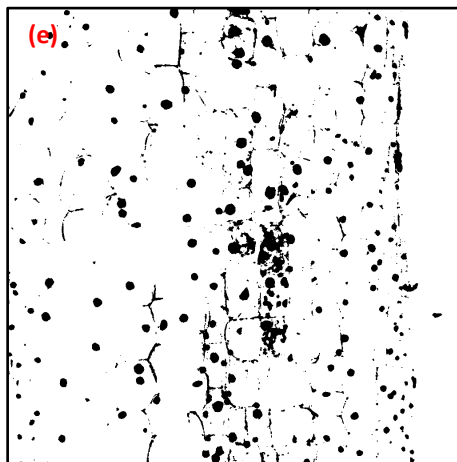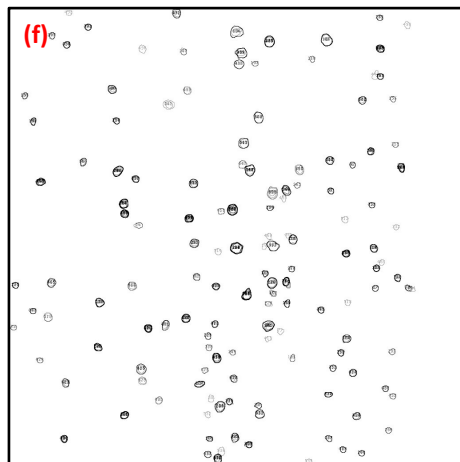

Supplement: Supplementary file 3 — Schematic illustration showing how nuclei are detected by the automated digital image analysis method (Round Surface Detector). (a) Original DAPI-stained image is spatially calibrated. (b) Result of 8-bit conversion followed by adjustement of brightness and contrast of each frame with final 3D rendering. (c) Result after the increase of image quality by subtracting background and calibrating the threshold in order to obtain a mask. (d) Mask based on threshold is before blurred with a smooth (e) then adjusted by filling holes and separating linked objects with a watershed in order to define the nuclear borders. (f) Selection from E loaded into the original image where the surfaces are measured using a high circularity index (0,7–1). Scale bars = 50 μm (PDF 1789 kb) [file 12870_2019_1791_MOESM3_ESM.pdf]

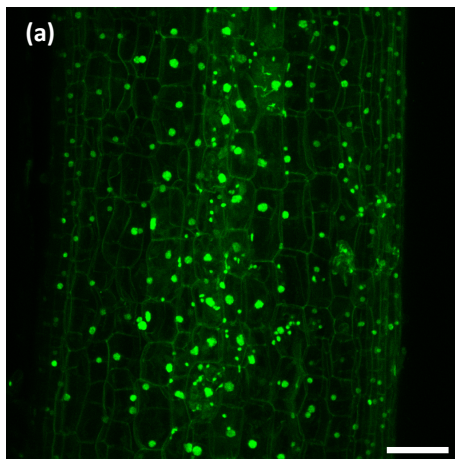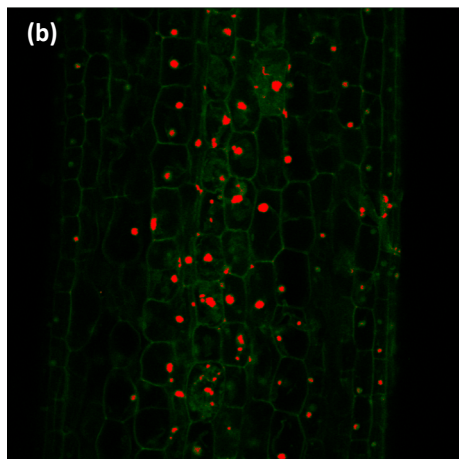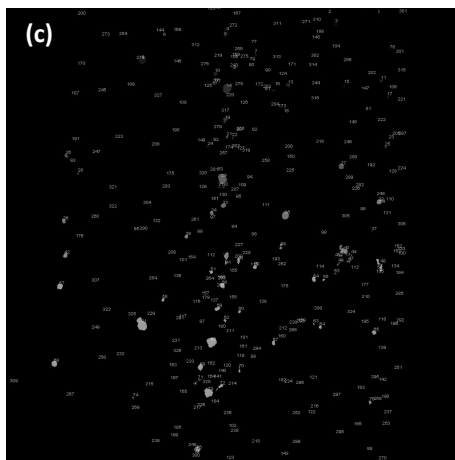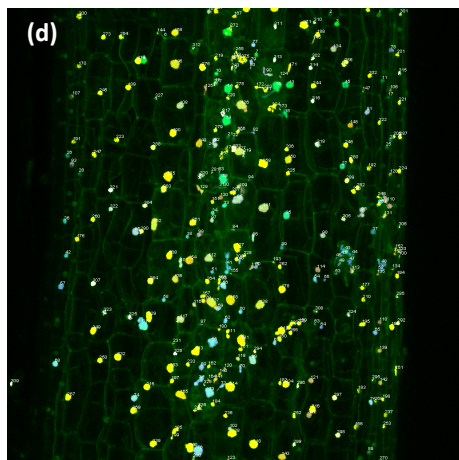

Supplement: Supplementary file 5 — Schematic illustration showing how nuclear volumes are detected using 3D Object Counter. (a) Original DAPI-stained image is spatially calibrated and adjusted in brightness and contrast. (b) A threshold was applied to define the limit intensity value separating the voxels between background voxels (intensities below the selected value) and objects voxels. (c) A 3D surface map containing only nuclear voxels is generated and the volume of each nucleus is calculated. (d) A merge between original z-stacks and 3D surface map is created. Scale bars = 50 μm (PDF 2518 kb) [file 12870_2019_1791_MOESM5_ESM.pdf]

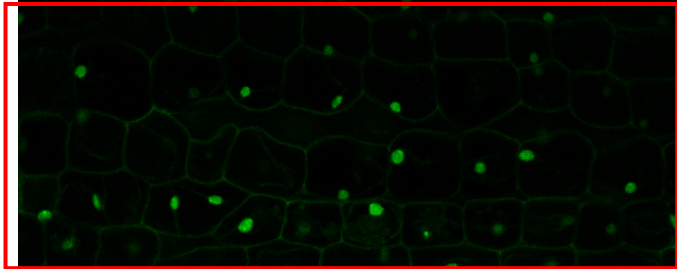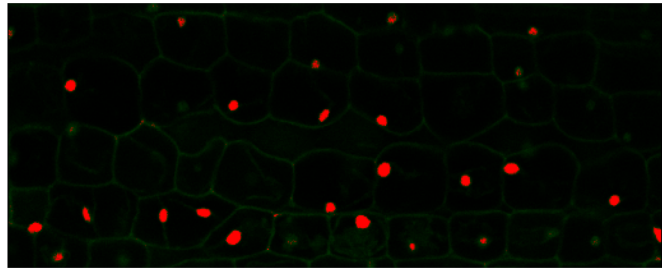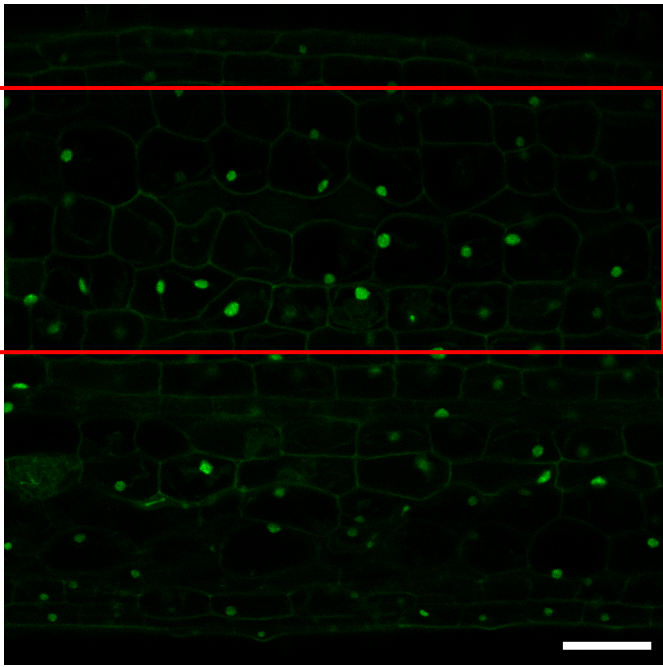

Supplement: Supplementary file 6 — Nuclear volume analysis of M. truncatula root cortex. Schematic illustration showing how nuclear volume detection is restricted to the cortical tissue by cropping (red box and right panel) the original z-stacks (left panel). The image dataset was then analysed using Fiji plugin 3D Object Counter, discriminating between the brighter nuclear voxels and the darker background. Scale bars = 50 μm (PDF 954 kb) [file 12870_2019_1791_MOESM6_ESM.pdf]

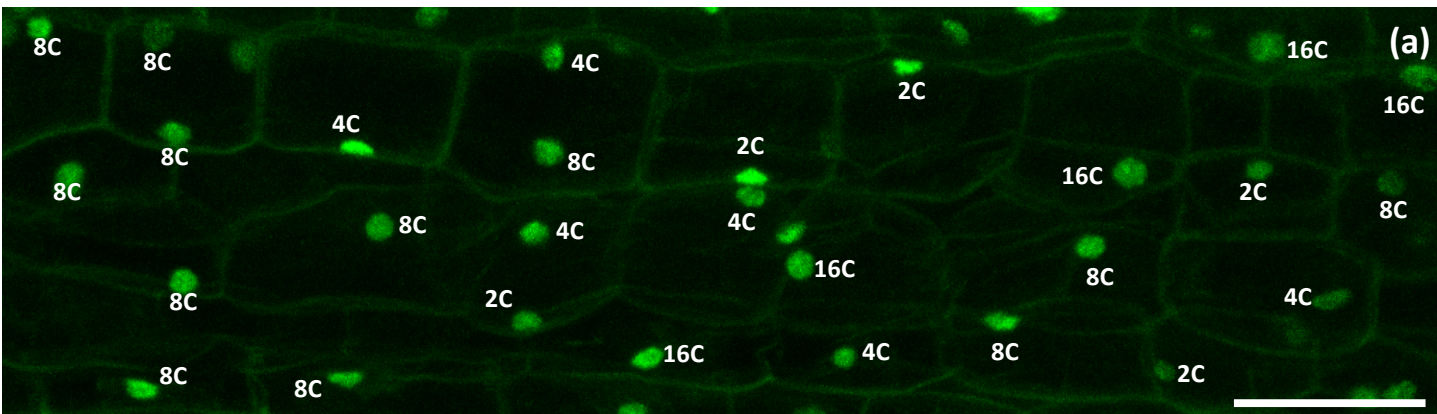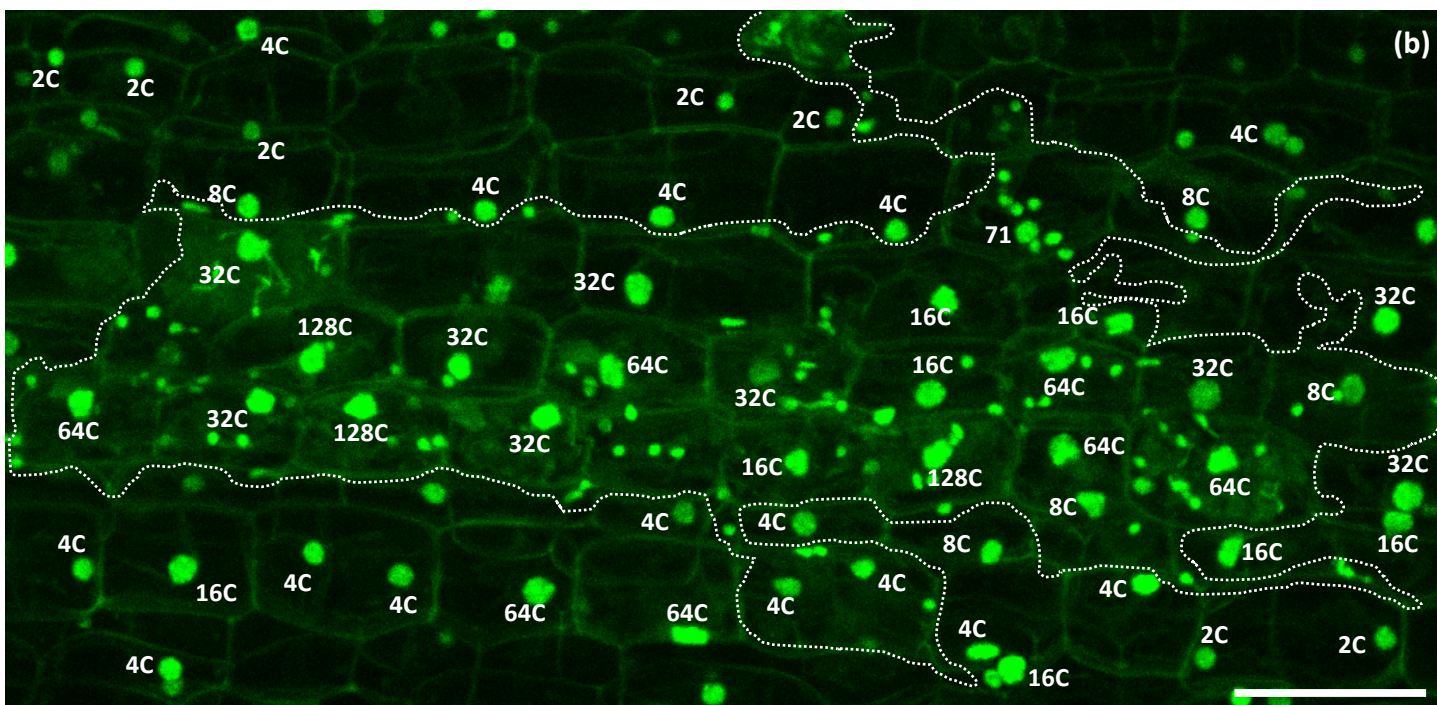

Supplement: Supplementary file 8 — Maps of putative ploidy in the cortex of uninoculated and mycorrhizal roots of M. truncatula. Based on the Sturges rule analysis of all nuclear volume measures derived by 3D Object Counter (Additional file 7), four and eight ploidy classes were respectively identified in uninoculated (a) and mycorrhizal (b) sections. Panels show representative images. Scale bars = 50 μm (PDF 671 kb) [file 12870_2019_1791_MOESM8_ESM.pdf]
