# Supplementary material for: Size matters: three methods for estimating nuclear size in mycorrhizal roots of Medicago truncatula by image analysis
Source: BMC Plant Biol. 2019 May 4;19:180. doi: 10.1186/s12870-019-1791-1 (PMC6500585; doi:10.1186/s12870-019-1791-1)
Supplement: Supplementary file 7 — Putative ploidy classes based on Sturges analysis of nuclear volume measures. All nuclear volumes measured with 3D Object Counter in 375x375x45μm z-stack projections from M. truncatula uninoculated (6 root sections, 594 nuclei) and mycorrhizal (6 root sections, 893 nuclei) ROC segments were clustered according to Sturges’ rule into 25 μm3 wide classes. Four classes were identified in uninoculated and eight in mycorrhizal sections; size limits and average volume ± standard deviation are presented for each class (PDF 29 kb) [file 12870_2019_1791_MOESM7_ESM.pdf]

Putative ploidy levels	Sturges Classes	Mean values Wt Ctr	Mean values Wt Myc
2C	20-45 μm³	30.3 ± 6.9	29.6 ± 7
4C	45-70 μm³	54.6 ± 7.1	55.2 ± 6.9
8C	70-95 μm³	77.6 ± 7	81 ± 6.9
16C	95-120 μm³	107 ± 9.6	105.9 ± 7.1
32C	120-145 μm³		131.8 ± 7.9
64C	145-170 μm³		155.3 ± 7.6
128C	170-195 μm³		181 ± 4.2
256C	195-220 μm³		210.2 ± 9.3
